# Biomimetic Cross-Scale Feature Recalibration with Axis-Decompositional Positional Embedding for Architectural Floor Plan Parsing

**DOI:** 10.3390/biomimetics11080581

**Published:** 2026-08-13

**Authors:** Jinting Zhou, Ruiyu Gao, Shijie Zhou, Fengli Zhang

**Affiliations:** School of Information and Software Engineering, University of Electronic Science and Technology of China, Chengdu 610054, China; 202112090805@std.uestc.edu.cn (J.Z.); 202322090502@std.uestc.edu.cn (R.G.); sjzhou@uestc.edu.cn (S.Z.)

**Keywords:** biomimetics, bio-inspired neural network, architectural floor plan parsing, semantic segmentation, cross-scale feature recalibration, positional embedding, building information modeling

## Abstract

Automatic semantic parsing of architectural floor plans provides a two-dimensional semantic layer for building information modeling (BIM)-related workflows, intelligent plan checking, renovation, and large-scale drawing management. Unlike natural images, floor plans contain sparse textures, dense linework, small architectural symbols, and strong axis-aligned geometric regularities. These properties make conventional segmentation networks vulnerable to small-symbol dilution during downsampling, noisy skip-feature fusion, and fragmented predictions along long wall boundaries. Inspired by principles of hierarchical visual processing, selective attention, and spatial encoding, this study presents PCP-Net, an end-to-end Planar Component Parsing Network for room, icon, and boundary-aware floor plan parsing. PCP-Net uses a Grouped Residual Encoder to extract multi-scale local patterns, a Cross-Scale Feature Recalibration (CSFR) pipeline to recalibrate skip features through Adaptive Channel Gating, Spatial Response Amplification, and Axis-Decompositional Positional Embedding, and a Structural Gradient Propagation branch to provide training-time boundary regularization. Experiments on CubiCasa5K and three external datasets (R3D, CVC-FP, and ROBIN) evaluate PCP-Net under in-domain and zero-shot cross-domain protocols. On CubiCasa5K, PCP-Net attains 71.3% mIoU, 90.1% overall accuracy, and 78.9% mean accuracy; in the current ablation setting, ADPE improves mIoU by 0.8 percentage points over the ADPE-ablated configuration. These results indicate that cross-scale feature recalibration with axis-aware positional cues can improve floor plan semantic parsing within the evaluated datasets and pixel-level metrics, while downstream BIM generation still requires additional vectorization, topology graph construction, and attribute extraction.

## 1. Introduction

The automatic parsing of architectural floor plans has long stood at the intersection of computer vision and architectural informatics. Early systems relied on hand-crafted geometric rules, but the field shifted decisively with the advent of deep learning: Dodge et al. pioneered CNN-based floor plan parsing [1], and Kalervo et al. established CubiCasa5K as a standard benchmark for multi-task layout analysis [2]. Subsequent work has extended this line to raster-to-vector conversion [3], boundary-guided recognition [4], and generative layout refinement [5], underscoring the practical demand for intelligent floor plan understanding.

These advances rest on the broader progress of semantic segmentation. Shelhamer et al. proposed FCN, replacing fully connected layers with convolutions to enable end-to-end pixel-wise prediction [6]; Ronneberger et al. subsequently introduced the U-Net encoder-decoder architecture with symmetric skip connections, which has become a staple for dense prediction tasks [7]. Deeper backbones such as ResNet [8] and ResNeXt [9] further enhanced hierarchical feature extraction, while attention mechanisms refined feature quality through channel recalibration in SENet [10] and sequential channel-spatial gating in CBAM [11]. Coordinate attention has emerged as an effective means to embed spatial positioning [12]. Boundary-aware designs such as Gated-SCNN [13] further demonstrate that architectural layouts demand more than generic pixel classification. However, most generic segmentation and attention modules were developed for natural-image scenes, where texture, object appearance, and category-level context often dominate. Architectural floor plans instead rely on sparse linework, axis-aligned geometry, closed contours, and room adjacency, which calls for mechanisms tied more explicitly to floor-plan-specific geometric priors.

Yet existing methods largely treat floor plans as ordinary scene images, ignoring the rigid structural constraints that govern valid architectural layouts. Three specific challenges arise. First, deep networks suffer semantic dilution when confronted with diverse room categories and complex spatial arrangements. Second, standard convolutions struggle to align wall boundaries precisely, even though edge fidelity strongly affects downstream room separation and layout interpretation. Third, models frequently produce fragmented boundary responses when transferred across drawing styles, despite promising advances in domain adaptation [14,15]. Lightweight deployment is also essential for real-world design tools [16].

The proposed design is also informed by recent research in biological and computational vision, including goal-driven deep learning models of sensory cortex [17], deep neural networks as a framework for modeling biological vision [18], convolutional neural networks as models of the visual system [19], feature binding [20], and saliency-based attention [21]. We use these principles as bio-inspired design analogies, not as evidence that PCP-Net reproduces biological visual mechanisms.

Guided by this bio-inspired and geometry-aware perspective, we propose PCP-Net (Planar Component Parsing Network), a unified encoder–decoder framework that exploits structural inductive biases inherent to architectural drawings: the dominance of horizontal and vertical axis-aligned structures, the prevalence of small architectural symbols, and the importance of continuous wall boundaries for interpretable room layouts.

The main contributions are summarized as follows:

- A grouped residual encoder that extracts multi-scale and diverse local patterns through partitioned channel groups, preserving fine-grained line details and global layout context across hierarchical stages.
- A cross-scale feature recalibration pipeline that purifies skip features before decoder fusion by suppressing noisy channels, amplifying salient spatial locations, and recovering axis-aware positional cues weakened by downsampling.
- A structural gradient propagation branch that provides training-time boundary regularization to reduce fragmented wall responses.
- An empirical evaluation on CubiCasa5K, R3D, CVC-FP, and ROBIN under in-domain and zero-shot cross-domain protocols. Relative to the bare encoder–decoder, the complete pipeline improves mean IoU by 18.4 percentage points. Compared with PCP-Net without ADPE, axis-aware positional modulation further contributes 0.8 mean IoU, 1.44 room mIoU, 0.76 icon mIoU, and 0.7 overall accuracy.

## 2. Related Work

### 2.1. Floor Plan Analysis and Datasets

The analysis of architectural floor plans has evolved from early rule-based systems to contemporary deep learning approaches. Early methods relied on hand-crafted geometric reasoning for room detection and symbol extraction [22]. The subsequent adoption of statistical learning frameworks led to the CVC-FP dataset and associated structural segmentation baselines [23,24], later refined by specialized recognition modules tailored to architectural drawings [25]. The release of CubiCasa5K [2] marked a significant inflection point, enabling end-to-end CNN-based parsing that outperformed traditional pipelines [1].

Beyond segmentation, recent research has increasingly addressed vectorization and reconstruction. Liu et al. proposed a raster-to-vector framework for structured floor plan transformation [3]. For 3D-to-layout reconstruction, FloorNet processes raw scans through a unified voxel-based architecture [26], while Floor-SP formulates reconstruction as a sequential room-wise shortest-path problem [27]. Boundary-guided recognition has been advanced through room-boundary attention mechanisms [4], and generative modeling has produced adversarial layout refinement networks capable of professional-quality designs [5]. Complementing these, Jang et al. explored indoor reconstruction directly from floor plan images [28], and Wu et al. investigated data-driven interior plan generation for residential buildings [29]. Despite these advances, existing methods predominantly treat floor plan parsing as a generic segmentation task, leaving boundary continuity and layout-specific structural constraints underexplored.

### 2.2. Semantic Segmentation Architectures and Attention Mechanisms

The foundation of modern semantic segmentation was established by FCN, which replaced fully connected layers with convolutions to enable pixel-wise prediction [6]. Ronneberger et al. subsequently introduced U-Net, a symmetric encoder-decoder network with skip connections that remains influential for dense prediction tasks [7]. To capture richer contextual information, Chen et al. developed DeepLab with atrous convolution and spatial pyramid pooling [30], while Zhao et al. proposed PSPNet to aggregate multi-scale features through pyramid pooling modules [31].

For real-time scenarios, Yu et al. presented BiSeNet, which decouples semantic and spatial processing through bilateral pathways [32]; its successor, BiSeNetV2, improves accuracy and speed via guided feature aggregation [33]. Yuan et al. introduced object-contextual representations to model long-range dependencies without excessive computational overhead [34]. More recently, the Transformer paradigm has reshaped the field: Zheng et al. proposed SETR, formulating segmentation as a sequence-to-sequence translation task [35]; Xie et al. introduced SegFormer, a hierarchical Transformer with a lightweight decoder [36]; and universal architectures such as Mask2Former [37] and Panoptic SegFormer [38] have demonstrated the efficacy of masked attention for diverse segmentation formats.

These architectures depend critically on backbone networks for hierarchical feature extraction. He et al. established the foundation with ResNet, using residual connections to enable training of very deep networks [8]. Xie et al. introduced ResNeXt, which improved representational capacity through grouped convolutions with increased cardinality [9]. Gao et al. proposed Res2Net, constructing multi-scale representations within individual residual blocks [39], while Chollet et al. pushed the efficiency frontier with Xception through depthwise separable convolutions [40]. Wang et al. proposed HRNet to maintain high-resolution representations throughout the network rather than recovering resolution through upsampling [41], and Radosavovic et al. systematized the design of efficient architectures through RegNet, discovering simple design spaces with strong accuracy-efficiency trade-offs [42].

To refine the features extracted by these backbones, attention mechanisms have become essential. Hu et al. pioneered channel attention with SENet, which adaptively recalibrates channel-wise responses through global pooling and gating [10]. Woo et al. extended this with CBAM, applying sequential channel and spatial attention to model both “what” and “where” to emphasize [11]. For long-range context, Wang et al. proposed Non-local networks, which compute self-attention over the entire feature map at substantial computational cost [43]. Subsequent works have sought more efficient alternatives: Huang et al. introduced CCNet, using criss-cross attention to reduce complexity from quadratic to linear [44]; Hou et al. proposed Coordinate Attention, embedding positional information into channel attention through factorized spatial pooling [12]; Wang et al. presented ECA-Net, avoiding dimensionality reduction in channel gating via 1D convolutions [45]; Li et al. developed SKNet for adaptive kernel selection across multiple receptive fields [46]; Fu et al. proposed DANet to model both channel and spatial dependencies through a dual attention mechanism [47]; and Park et al. explored BAM for bottleneck-stage feature refinement [48]. Although these mechanisms have improved general scene segmentation, most were designed around natural-image appearance statistics rather than sparse linework, axis-aligned boundaries, small symbols, and room-adjacency relations. Floor plan parsing instead requires recalibration that preserves horizontal and vertical positional cues before skip fusion and supports continuous boundary responses. In this setting, PCP-Net differs from generic attention modules by applying channel and spatial recalibration to skip features and restoring axis-wise positional cues before decoder fusion, while the boundary branch provides training-time regularization for boundary responses.

### 2.3. Bio-Inspired and Geometry-Aware Inductive Biases

Bio-inspired vision studies, including goal-driven deep learning models of sensory cortex [17], deep neural networks as a framework for biological vision [18], convolutional networks as models of the visual system [19], feature binding [20], and saliency-based attention [21], provide useful analogies for hierarchical, selective, spatially organized parsing. For architectural drawings, the relevant lesson is not biological replication but the need for task-specific inductive biases. PCP-Net therefore translates these principles into grouped multi-scale encoding, channel and spatial recalibration, axis-wise positional embedding, and a training-time boundary branch. This formulation keeps the bio-inspired motivation connected to sparse symbols, rectilinear walls, and boundary-dependent room separation without overstating biological equivalence.

### 2.4. Boundary-Aware Structure and Cross-Domain Robustness

Accurate boundary delineation is particularly critical for floor plan segmentation, as wall boundaries strongly affect room separation and spatial adjacency. Takikawa et al. proposed Gated-SCNN, which explicitly models boundaries through a dual-branch architecture where a gating mechanism fuses semantic and edge prediction streams [13]. Liu et al. introduced self-calibrated convolutions to enhance receptive field adaptability, thereby improving fine-grained structure capture without increasing network depth [49]. Wu et al. further highlighted the importance of structural constraints in data-driven interior layout generation, indicating that closed rooms and connected walls are important complements to per-pixel accuracy in layout interpretation [29].

Structural consistency is equally important when models are transferred across domains. Floor plan images may originate from CAD renderings, rasterized bitmaps, or scanned documents, each exhibiting distinct stylistic characteristics. To bridge this gap, Ganin et al. proposed DANN, using adversarial training with a gradient reversal layer to learn domain-invariant representations [14]. Tsai et al. introduced AdaptSegNet, which aligns the structured output space of segmentation networks across domains [15]. Yang et al. presented FDA, a surprisingly effective approach that swaps low-frequency spectra between source and target images in the Fourier domain [50]. Hoffman et al. developed CyCADA, leveraging cycle consistency to preserve semantic content during cross-domain transfer [51]. These techniques improve generalization, but they usually address domain shift through representation or image-level alignment rather than explicit architectural constraints. For floor plans, such constraints are better framed as boundary continuity, reduced fragmentation, and plausible room separation, all of which are important for interpretable layout parsing but require task-specific modeling.

### 2.5. Lightweight Network Design

For deployment in design software or resource-constrained environments, computational efficiency is paramount. Howard et al. introduced MobileNet, which factorizes standard convolution into depthwise and pointwise operations to reduce both parameters and computational cost [52]. Tan et al. proposed EfficientNet, systematically scaling network depth, width, and resolution through compound coefficients to optimize accuracy-efficiency trade-offs [53]. Han et al. presented GhostNet, generating additional feature maps from inexpensive operations to reduce redundancy while preserving representational capacity [16]. Balancing these efficiency gains with the boundary precision and reduced boundary fragmentation needed for floor plan parsing remains an open design issue.

## 3. Methodology

### 3.1. Overall Architecture

**Figure 1:**
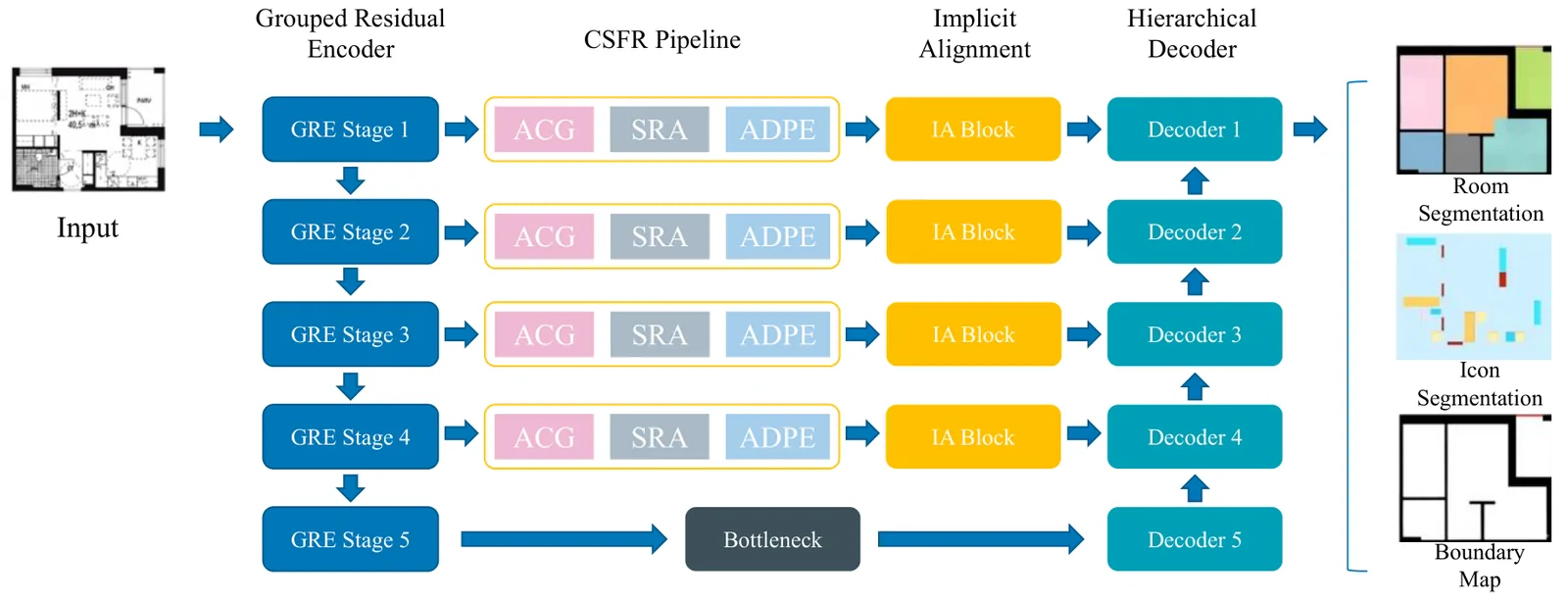
Overall architecture of PCP-Net. The CSFR pipeline comprises Adaptive Channel Gating (ACG), Spatial Response Amplification (SRA), and Axis-Decompositional Positional Embedding (ADPE). Recalibrated skip features are fused with decoder features via a lightweight implicit alignment block before multi-head prediction.

Given an input floor plan image $I\in R^{H\times W\times 3}$, PCP-Net predicts three complementary outputs: a room semantic map, an architectural icon map, and a structural boundary map used only during training. The network is organized into five stages: (1) input normalization and resizing to a fixed spatial resolution; (2) grouped residual encoding to extract hierarchical features $\left\{E_{1},E_{2},E_{3},E_{4},E_{5}\right\}$; (3) cross-scale feature recalibration (CSFR) to recalibrate skip connections before decoder fusion; (4) hierarchical decoding with multi-resolution upsampling; and (5) multi-head prediction. The overall architecture is illustrated in Figure 1.

Shallow encoder features ($E_{1}$, $E_{2}$) preserve fine-grained line and symbol details, while deep features ($E_{4}$, $E_{5}$) encode room-level layout context. The CSFR pipeline transforms each raw skip link into a recalibrated representation that suppresses background clutter and highlights structural cues. Rather than direct concatenation, these recalibrated features are fused with upsampled decoder features via a lightweight implicit alignment block, which mitigates misalignment artifacts across scales. The room and icon branches share the same decoder but emit separate logits, whereas the boundary branch provides auxiliary structural regularization. During deployment, the boundary head can be discarded.

### 3.2. Grouped Residual Encoder

Architectural drawings exhibit diverse local patterns ranging from regular geometric walls to irregular symbols, which a monolithic convolutional filter inadequately captures. PCP-Net adopts a Grouped Residual Encoder, in which feature transformations are partitioned into *G* parallel channel groups and aggregated via residual shortcuts.

For a feature map $X\in R^{H\times W\times C}$ at a given residual stage, we project it into a bottleneck representation and partition the channels into *G* groups. Let $X^{\left(i\right)}$ denote the *i*-th channel partition and $T_{i}\left(\cdot \right)$ the corresponding grouped transformation implemented by a stack of 3×3 convolutions. The grouped residual block is formulated as

(1) $$Y=X+{\mathrm{Conv}}_{1\times 1}\left(\left[T_{1}\left(X^{\left(1\right)}\right);\;T_{2}\left(X^{\left(2\right)}\right);\;\ldots ;\;T_{G}\left(X^{\left(G\right)}\right)\right]\right),$$

where [·;·] denotes channel concatenation and the final 1×1 projection restores the output dimension to match *X*. This design reduces inter-group dependencies and model complexity while increasing the diversity of learned local patterns. The parameter count of a grouped convolution with kernel size *k*, input channels $C_{\mathrm{in}}$, output channels $C_{\mathrm{out}}$, and *G* groups is

(2) $$P_{\mathrm{group}}=\frac{k^{2}C_{\mathrm{in}}C_{\mathrm{out}}}{G},$$

in contrast to the standard convolution

(3) $$P_{\mathrm{std}}=k^{2}C_{\mathrm{in}}C_{\mathrm{out}}.$$

Shallow stages maintain high spatial resolution to preserve narrow wall openings and small symbols, whereas deeper stages progressively enlarge the receptive field to capture room-level context. The effective receptive field at layer *l* follows the recursive relation

(4) $$r_{l}=r_{l-1}+\left(k_{l}-1\right)\prod_{j=1}^{l-1}s_{j},$$

where $k_{l}$ and $s_{j}$ denote the kernel size and stride, respectively. This design balances fine-grained symbol discrimination with global layout understanding.

### 3.3. Cross-Scale Feature Recalibration Pipeline

Standard encoder–decoder architectures fuse skip connections by directly concatenating high-resolution encoder features with upsampled decoder features. For floor plans, this naive fusion injects background clutter, scanning artifacts, and redundant line responses into the decoding stream. The CSFR pipeline addresses this by recalibrating each skip feature before decoder fusion, comprising three sequential stages: Adaptive Channel Gating (ACG), Spatial Response Amplification (SRA), and Axis-Decompositional Positional Embedding (ADPE).

#### 3.3.1. Adaptive Channel Gating

For a skip feature $F\in R^{H\times W\times C}$, ACG learns a channel-wise gating vector from two complementary global descriptors. Average pooling captures the global response intensity indicative of background dominance, while max pooling preserves salient structural activations corresponding to walls and symbols. The channel attention map is computed as

(5) $$M_{c}\left(F\right)=\sigma \left(\mathrm{MLP}\left(\mathrm{AvgPool}(F)\right)+\mathrm{MLP}\left(\mathrm{MaxPool}(F)\right)\right),$$

where σ denotes the sigmoid function and MLP is implemented by a two-layer bottleneck with a channel reduction ratio. The recalibrated feature is obtained by channel-wise scaling:

(6) $$F_{c}=M_{c}\left(F\right)⊙F.$$

ACG therefore suppresses noisy or background-dominated channels and amplifies those corresponding to walls, openings, and architectural symbols.

#### 3.3.2. Spatial Response Amplifier

Building upon the channel-gated feature $F_{c}$, SRA emphasizes salient spatial locations. Two channel-wise spatial descriptors are generated by compressing the channel dimension: an average-intensity map encoding structural density and a max-activation map highlighting edge and symbol peaks. These are concatenated along the channel dimension and processed by a large-kernel convolution to produce a spatial attention map:

(7) $$M_{s}\left(F_{c}\right)=\sigma \left({\mathrm{Conv}}_{7\times 7}\left([{\mathrm{AvgPool}}_{c}\left(F_{c}\right);\;{\mathrm{MaxPool}}_{c}\left(F_{c}\right)]\right)\right),$$

where ${\mathrm{AvgPool}}_{c}$ and ${\mathrm{MaxPool}}_{c}$ denote spatial average- and max-pooling along the channel axis, yielding H×W×1 descriptors. The spatially amplified feature is

(8) $$F_{s}=M_{s}\left(F_{c}\right)⊙F_{c}.$$

The large kernel aggregates local structural context, helping to emphasize continuous walls, room enclosures, and openings while suppressing blank background regions.

#### 3.3.3. Axis-Decompositional Positional Embedding

Global pooling discards positional traces by collapsing spatial features into a single vector, which is particularly harmful for floor plans because horizontal walls, vertical partitions, room extents, and door openings are strongly coordinate-dependent. ADPE preserves axis-wise context by separately aggregating features along the spatial width and height.

Given the spatially amplified feature $F_{s}$, the horizontal and vertical position-sensitive descriptors are

(9) $$z_{h}\left(c,y\right)=\frac{1}{W}\sum_{x=1}^{W}F_{s}\left(y,x,c\right),\;z_{w}\left(c,x\right)=\frac{1}{H}\sum_{y=1}^{H}F_{s}\left(y,x,c\right),$$

where $z_{h}\in R^{C\times H}$ captures row-wise horizontal context and $z_{w}\in R^{C\times W}$ captures column-wise vertical context. Concatenating along the spatial axis and applying a shared 1×1 convolution yields

(10) $$f=\delta \left({\mathrm{Conv}}_{1\times 1}\left([z_{h},\;z_{w}]\right)\right)\in R^{C/r\times (H+W)},$$

where δ is a nonlinear activation and *r* is the reduction ratio. After splitting into horizontal and vertical components $f_{h}\in R^{C/r\times H}$ and $f_{w}\in R^{C/r\times W}$, axis-specific attention weights are generated by separate 1×1 convolutions:

(11) $$a_{h}\left(c,y\right)=\sigma \left({\mathrm{Conv}}_{1\times 1}^{h}\left(f_{h}\right)\right),\;a_{w}\left(c,x\right)=\sigma \left({\mathrm{Conv}}_{1\times 1}^{w}\left(f_{w}\right)\right),$$

which are broadcast to $R^{H\times W\times C}$ and multiplied:

(12) $$F_{p}\left(y,x,c\right)=F_{s}\left(y,x,c\right)\cdot a_{h}\left(c,y\right)\cdot a_{w}\left(c,x\right).$$

Here, $z_{h}$ encodes row-wise horizontal structure (e.g., ceiling alignments, horizontal corridors), while $z_{w}$ encodes column-wise vertical structure (e.g., vertical walls, stairwell shafts). Their bilinear modulation provides location-dependent channel weighting without sacrificing spatial traceability.

#### 3.3.4. Hierarchical Feature Fusion with Implicit Alignment

The recalibrated skip feature $F_{p}$ is fused with the upsampled decoder feature $D_{l+1}$ through a lightweight alignment block. Rather than direct concatenation, which may misalign structural cues across scales, we first project both features into a common representation space:

(13) $${\tilde{D}}_{l}={\mathrm{Conv}}_{3\times 3}\left(\mathrm{Up}(D_{l+1})\right),\;{\tilde{F}}_{l}={\mathrm{Conv}}_{1\times 1}\left(F_{p}\right).$$

A depthwise-separable convolutional block then fuses the aligned streams:

(14) $$A_{l}=\phi \left([{\tilde{D}}_{l};\;{\tilde{F}}_{l}]\right),\;D_{l}=\psi \left(A_{l}\right)⊙A_{l}+{\tilde{D}}_{l},$$

where ϕ compresses the concatenated features into *C* channels and ψ denotes a pointwise re-weighting gate with sigmoid activation. The residual connection ${\tilde{D}}_{l}$ preserves gradient flow, while the gate $\psi (A_{l})$ adaptively emphasizes structurally salient features.

### 3.4. Structural Gradient Propagation Branch

Despite recalibrated skip features, decoder predictions remain uncertain around wall interfaces, room corners, and narrow openings. We therefore introduce an SGP branch to provide training-time boundary regularization.

A binary boundary target B(S) is extracted from the ground-truth semantic mask *S* by identifying pixels whose semantic label differs from at least one neighbor in a local neighborhood, followed by mild dilation to match the boundary tolerance used during supervision. The boundary consistency loss encourages structural overlap between predicted and ground-truth boundaries:

(15) $$L_{\mathrm{bd}}={∥B\left(\hat{S}\right)-B\left(S\right)∥}_{1},$$

where $\hat{S}$ is the predicted semantic map and B(·) is the boundary extraction operator defined above. On the prediction side, $B(\hat{S})$ is derived from the predicted probability map via a confidence threshold to encourage discrete boundary alignment.

To further sharpen structural transitions, a gradient consistency loss penalizes discrepancies in the spatial gradients of the semantic maps:

(16) $$L_{\mathrm{grad}}=\sum_{p}{∥\nabla \hat{S}\left(p\right)-\nabla S\left(p\right)∥}_{1},$$

where ∇ denotes the spatial gradient operator applied to the one-hot encoded ground-truth labels via Sobel filtering.

During training, the predicted boundary confidence map $\hat{B}$ can also be used to refine semantic logits, directing model attention toward uncertain structural regions:

(17) $${\hat{S}}_{\mathrm{ref}}=\hat{S}⊙\left(1+\alpha \hat{B}\right),$$

where $\hat{S}$ denotes the pre-softmax semantic logits, $\hat{B}$ is the sigmoid-activated boundary confidence, and α is a learnable or fixed modulation factor. This refinement is applied only during training to strengthen the influence of boundary-supervised gradients on the shared encoder representations.

### 3.5. Loss Function

The total training objective combines region-level semantic fidelity with boundary-aware structural regularization:

(18) $$L_{\mathrm{total}}=\lambda_{\mathrm{ce}}L_{\mathrm{CE}}+\lambda_{\mathrm{dice}}L_{\mathrm{Dice}}+\lambda_{\mathrm{bd}}L_{\mathrm{bd}}+\lambda_{\mathrm{grad}}L_{\mathrm{grad}}.$$

The cross-entropy loss $L_{\mathrm{CE}}$ optimizes pixel-wise classification, while the Dice loss $L_{\mathrm{Dice}}$ improves region overlap against class imbalance. The boundary and gradient losses jointly sharpen boundary responses and reduce fragmented predictions. Multi-resolution supervision may be optionally attached to intermediate decoder stages to stabilize early optimization and preserve semantic anchors at different scales.

## 4. Experiments

### 4.1. Datasets and Metrics

The primary evaluation benchmark is CubiCasa5K [2], a large-scale floor plan dataset comprising room regions, architectural icons, and structural elements. The room branch encompasses eleven categories: Background (BG), Outdoor (OD), Wall (W), Kitchen (KT), Living Room (LR), Bedroom (BR), Bathroom (BH), Hallway (HW), Railing (RL), Storage (SG), and Garage (GG). The icon branch comprises eleven categories: Empty (EM), Window (WI), Door (DR), Closet (CT), Electrical Appliance (EA), Toilet (TO), Sink (SK), Sauna Bench (SB), Fireplace (FP), Bathtub (BT), and Chimney (CH). To assess zero-shot generalization, three external datasets are employed: R3D [4], CVC-FP [23], and ROBIN [54]. Models are trained on CubiCasa5K only and directly evaluated on the external datasets without target-domain fine-tuning. For cross-domain evaluation, only overlapping categories are retained, and unmapped classes are excluded from the corresponding mean IoU scores.

The evaluation metrics include overall accuracy (OA), mean accuracy (MA), mean intersection over union (mIoU), room mIoU, and icon mIoU. OA measures global pixel correctness; MA reflects class-balanced accuracy; and mIoU quantifies region overlap. Boundary F1 and Boundary IoU are reported where boundary-level evaluation is included and are treated as structural proxy metrics for wall and room-contour alignment.

### 4.2. Implementation Details

The model is implemented in PyTorch 2.8.0 with an input resolution of 512×512 and a batch size of 8. The optimizer is AdamW with an initial learning rate of $3\times 10^{-4}$, weight decay of $10^{-2}$, and betas of (0.9,0.999). The learning rate schedule uses 1000 warm-up iterations followed by polynomial decay with power 0.9 and a minimum learning rate of $10^{-6}$. Training proceeds for 80 k iterations, with validation performed every 1 k iterations. Automatic mixed precision and gradient clipping with a threshold of 1.0 are used to improve numerical stability. Data augmentation includes random flipping, rotation, and scaling. The three output heads predict room semantics, icon semantics, and binary boundaries.

### 4.3. Main Results and Comparison

**Figure 2:**
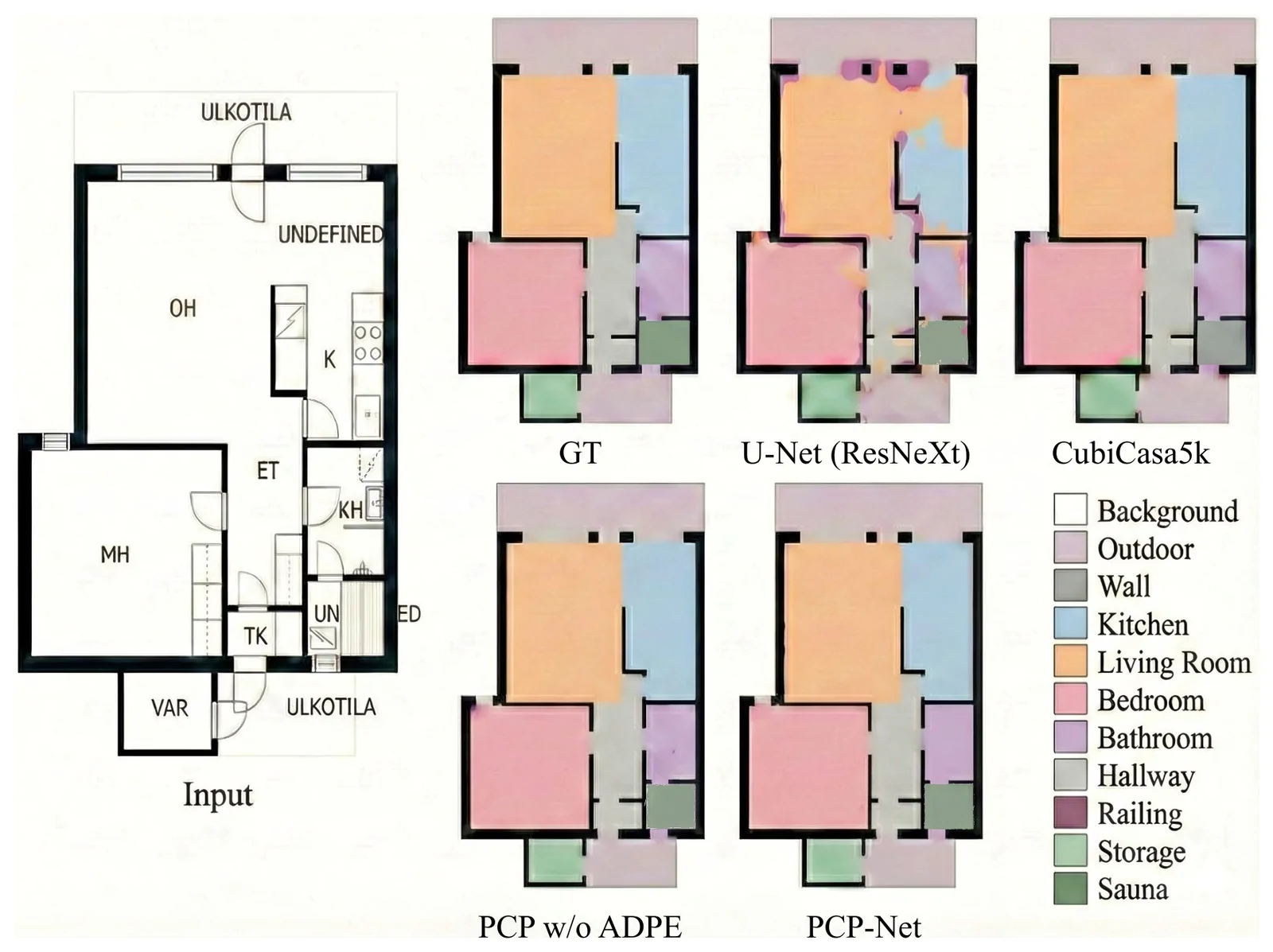
Qualitative comparison of room segmentation on the CubiCasa5k test set. From left to right: input floor plan, ground-truth room layout, U-Net with ResNeXt backbone prediction, CubiCasa5k baseline prediction, PCP-Net without ADPE, and full PCP-Net. The progressive introduction of grouped residual encoding and cross-scale feature recalibration substantially improves wall closure and reduces topological fragmentation in adjacent rooms and narrow corridors.

To evaluate the overall effectiveness of the proposed architecture, we conduct controlled comparisons on the CubiCasa5K test set under a shared evaluation protocol. Table 1 reports the comparison with representative floor-plan-specific and general semantic segmentation baselines. The contribution of each architectural component is analyzed in the ablation study (Section 4.5). Figure 2 supplements this quantitative analysis with qualitative comparisons against two additional reference points: a generic U-Net with ResNeXt backbone, which represents the standard encoder–decoder paradigm without any floor-plan-specific adaptation, and the CubiCasa5K baseline, which reflects the original task-specific training pipeline yet lacks the structural recalibration mechanisms proposed herein.

The bare grouped encoder produces severely fragmented room boundaries and frequently confuses functionally adjacent spaces, reflecting that standard grouped convolutions alone cannot capture the rigid geometric constraints inherent to architectural drawings. The intermediate configuration, which incorporates adaptive channel gating and spatial response amplification, recovers most region boundaries and reduces most category-level errors. The incremental contribution of ADPE appears numerically modest yet architecturally pivotal: it primarily refines long-range spatial dependencies, sharpening room corners and maintaining consistent hallway connectivity rather than merely boosting aggregated pixel-level accuracy. This pattern is consistent with the role of axis-aware positional cues in reducing boundary misalignment introduced by repeated downsampling, not as an additive feature of marginal utility.

Figure 2 illustrates the qualitative differences among these competing approaches, which are consistent with the quantitative results in Table 1. The U-Net with ResNeXt backbone captures the coarse room layout yet frequently merges adjacent spaces and misses narrow wall openings because its monolithic convolutional filters lack the diversity to handle varying wall widths and heterogeneous drawing conventions. The CubiCasa5K baseline improves region separation through task-specific training but still exhibits blurred boundaries between functionally adjacent rooms such as bedrooms and hallways, indicating that architectural parsing demands more than dataset-specific fine-tuning of generic architectures. The intermediate PCP-Net configuration without ADPE already produces crisp wall lines and correctly separated room enclosures, consistent with the contribution of channel and spatial recalibration to structural recovery. The full PCP-Net further strengthens the continuity of long wall segments and reduces spurious misclassification at T-junctions and corner turns, indicating that the complete pipeline effectively couples local symbol discrimination with global layout consistency.

### 4.4. Cross-Domain Generalization

All cross-domain experiments are conducted in a zero-shot setting: all models are trained only on the CubiCasa5K training split and directly evaluated on R3D, CVC-FP, and ROBIN without target-domain fine-tuning or domain adaptation. Because the external datasets adopt different annotation schemes, only overlapping room categories are included in the mean IoU calculation, and unmapped or background categories are excluded from the reported metrics. Table 2 reports the cross-domain quantitative results.

**Figure 3:**
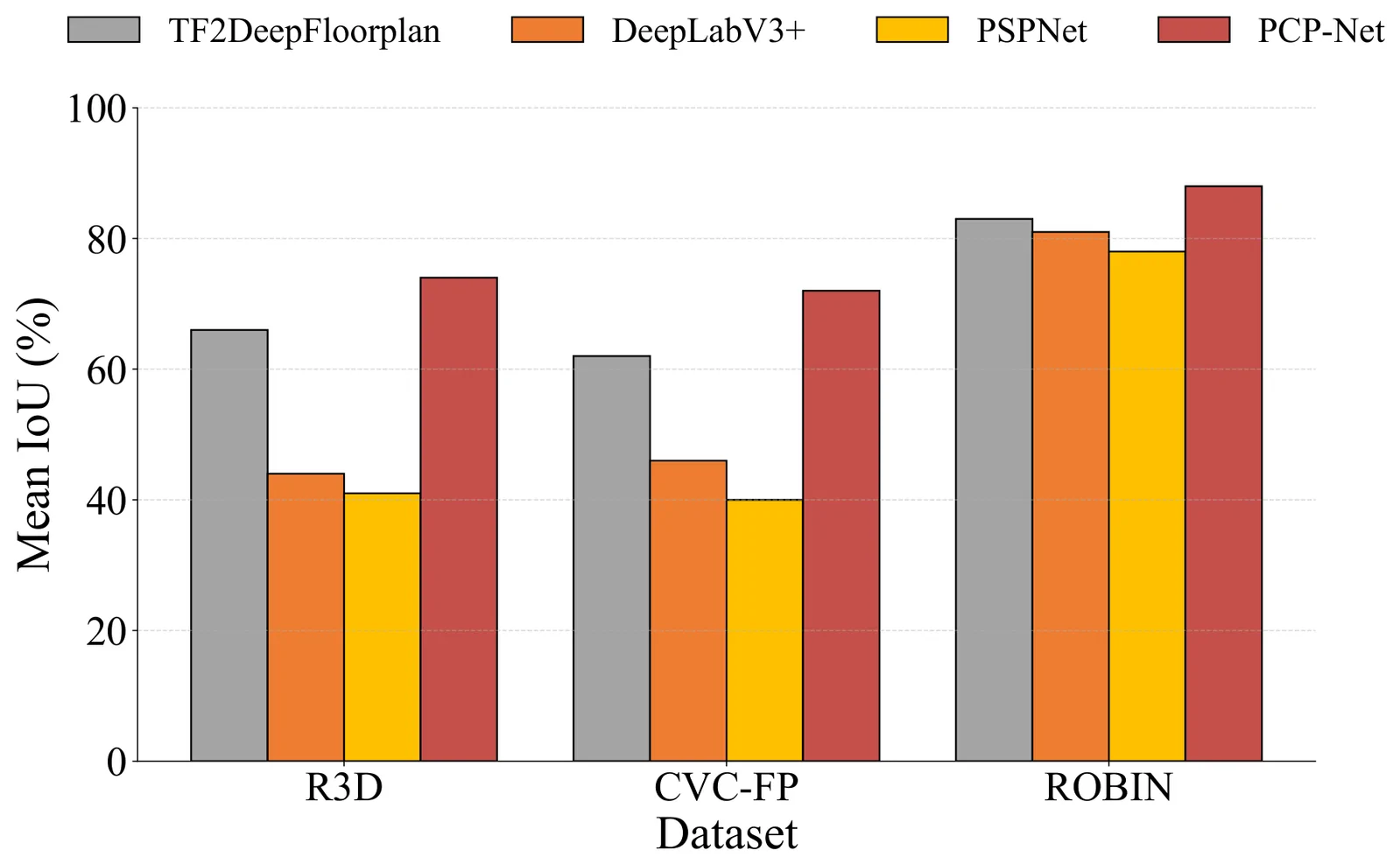
Cross-domain mean IoU comparison on R3D, CVC-FP, and ROBIN. PCP-Net consistently outperforms the competing methods across all three unseen domains.

Figure 3 aggregates the mean IoU comparisons across the three external datasets, showing a consistent performance margin of PCP-Net over the competing approaches under the evaluated protocol.

The cross-domain evaluation reveals that structural cues learned from CubiCasa5K transfer to the evaluated external datasets. On R3D, where Manhattan-style layouts dominate, the model achieves the strongest generalization because the orthogonal wall assumptions embedded in ADPE align closely with the target domain geometry. The intermediate configuration already outperforms competing methods by a substantial margin, underscoring that channel and spatial recalibration alone capture robust structural cues. The full PCP-Net further consolidates this advantage through axis-aware modulation, which appears beneficial for aligning walls that span multiple rooms in rectilinear configurations.

CVC-FP presents a more challenging scenario: scanned textures and low-contrast linework degrade boundary cues, yet PCP-Net maintains the highest recovery rate among all competitors by leveraging its spatial response amplification to suppress scanning noise. The performance gap between PCP-Net and its ADPE-ablated variant is most pronounced on the wall and doorway categories, where positional priors compensate for the missing high-frequency edge information that scanning artifacts typically attenuate.

ROBIN exhibits the largest stylistic departure from the training distribution, yet the model still preserves wall closure with minimal fragmentation, suggesting that the recalibration pipeline captures structural cues that transfer under the evaluated domain shifts rather than overfitting to specific rendering styles. The consistent margin between PCP-Net and its intermediate configuration across all three domains indicates that axis-aware positional cues provide a transferable inductive bias for architectural drawings, one that remains effective even when the input domain lacks the clean, high-contrast line renderings present in the training set.

**Figure 4:**
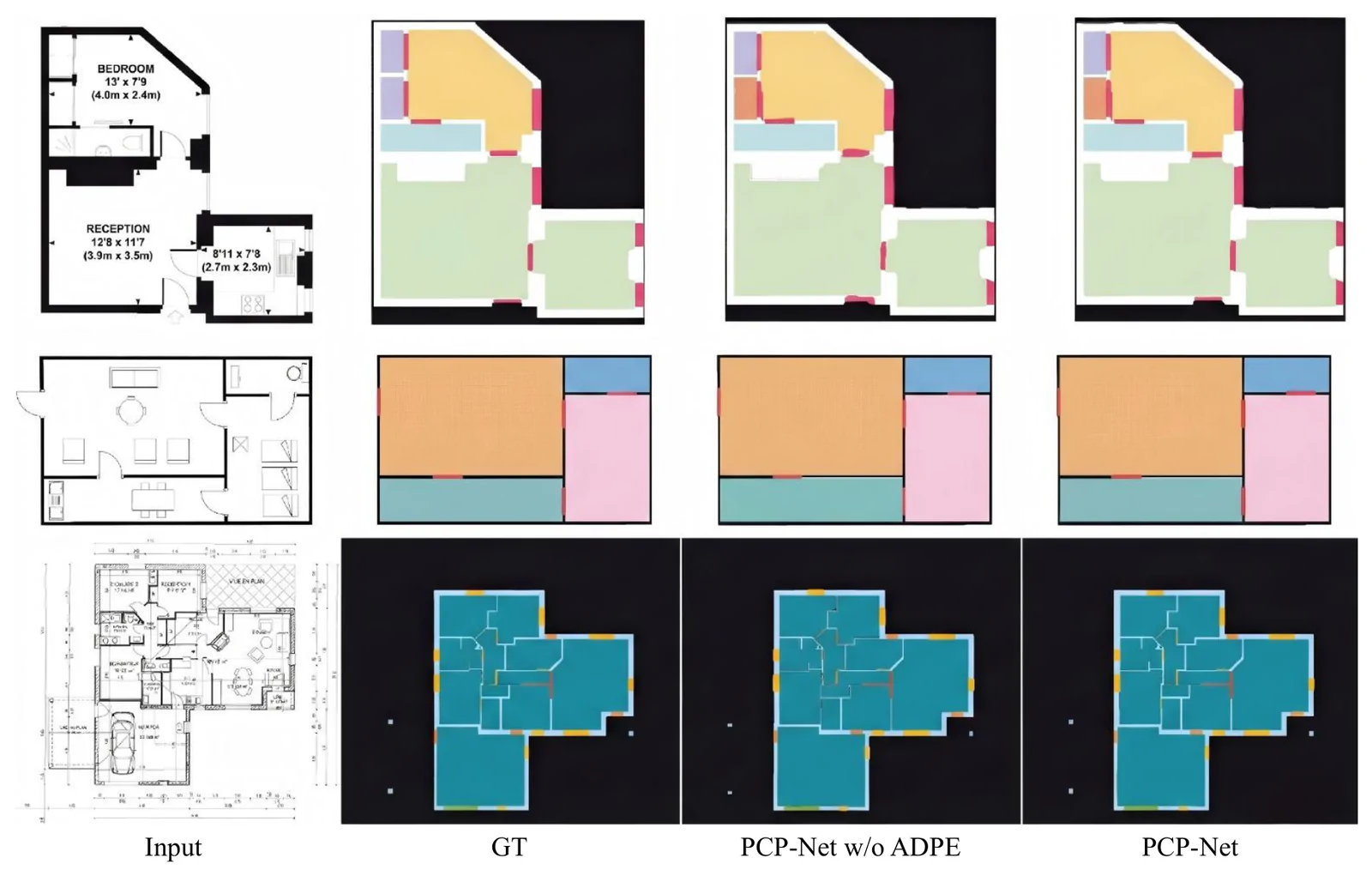
Zero-shot cross-domain qualitative results on R3D, CVC-FP, and ROBIN. The full PCP-Net preserves wall closure and room topology consistency under domain shift, whereas the intermediate configuration without ADPE exhibits more fragmented boundary responses and spurious room mergers.

Figure 4 illustrates these quantitative findings at the visual level. On R3D samples, the model correctly distinguishes living rooms from adjacent bedrooms even when wall lines are thin and partially occluded by furniture annotations, a behavior that generic segmentation frameworks struggle to replicate because they lack the explicit axis-aware modulation required for rectilinear alignment. For CVC-FP scans, the full PCP-Net suppresses the bleeding of wall labels into background regions that typically occurs when models rely solely on texture cues, suggesting that the proposed recalibration mechanisms privilege structural geometry over surface appearance. The ROBIN cases demonstrate the most dramatic improvement: without ADPE, the prediction frequently breaks corridor walls into disconnected segments, whereas the full model maintains continuous wall lines across the entire span, thereby suggesting that the positional embedding module is particularly critical when the input domain lacks the clean, high-contrast line renderings present in the training set.

### 4.5. Ablation and Structural Analysis

To isolate the contribution of each architectural component, a controlled ablation study is conducted on the CubiCasa5K test set. The baseline contains the grouped encoder and hierarchical decoder without feature recalibration while retaining the SGP boundary branch. The +SRA and +ACG configurations separately evaluate spatial and channel recalibration, respectively; the combined ACG+SRA configuration is denoted as PCP-Net w/o ADPE. The w/o SGP configuration removes only the SGP branch from the full model. All configurations are trained with three random seeds; we report the mean ± standard deviation and include Boundary F1 and Boundary IoU as structural proxy metrics. Table 3 reports the overall ablation results.

**Figure 5:**
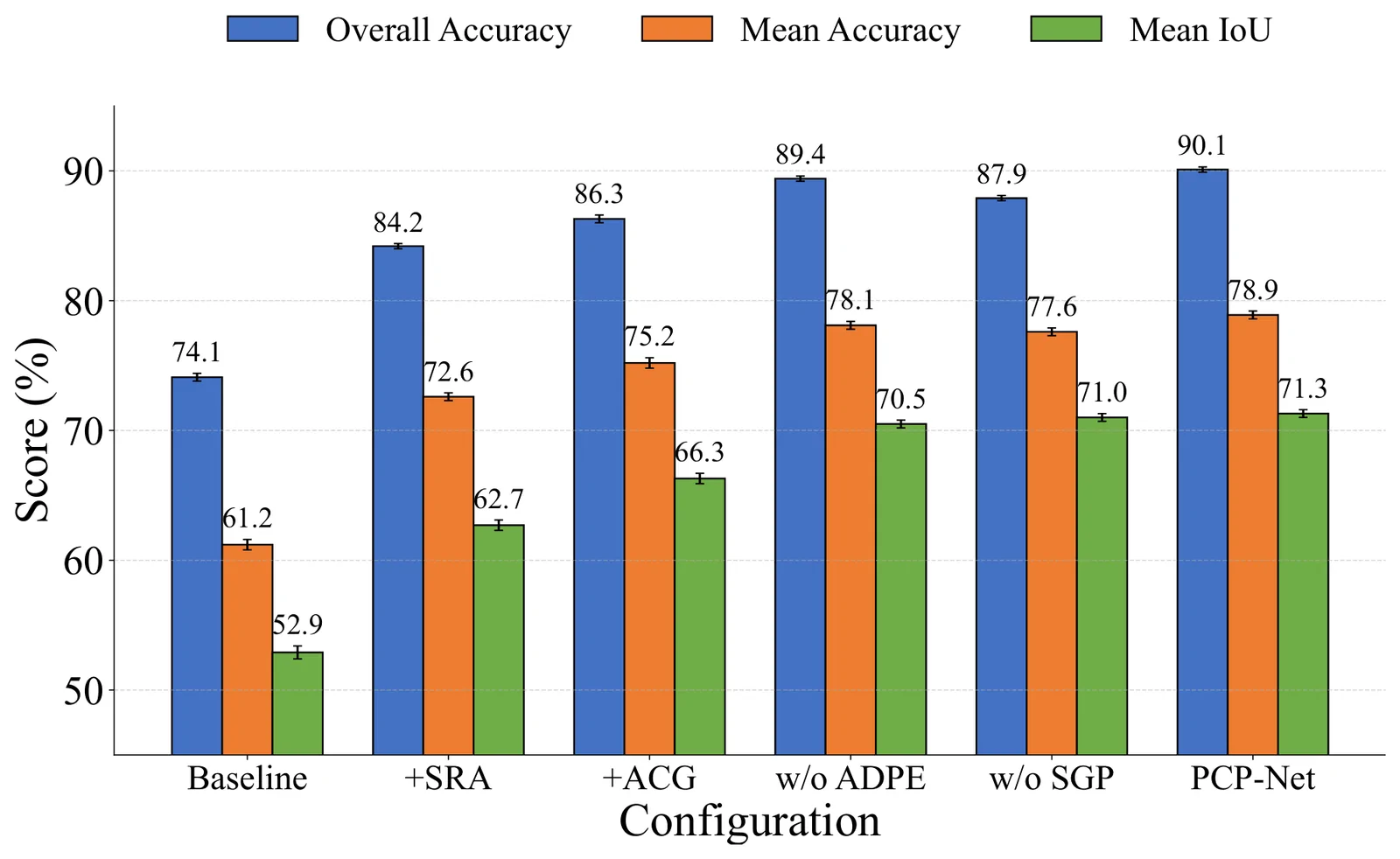
Overall ablation comparison across OA, MA, and mIoU (mean ± std over three seeds).

**Figure 6:**
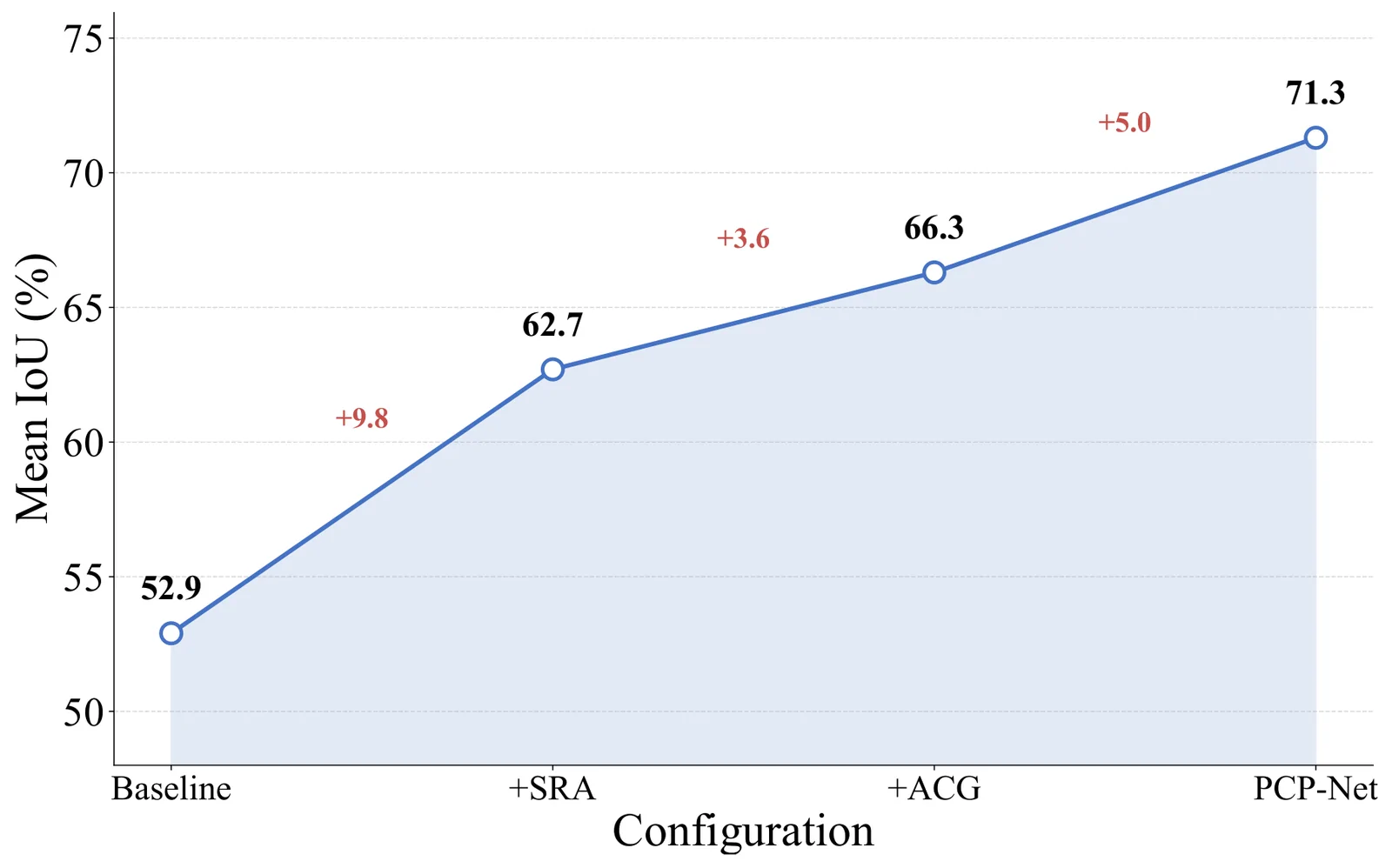
mIoU comparison across ablation configurations (mean ± std over three seeds).

Figure 5 and Figure 6 visualize the quantitative comparison across ablation configurations with standard-deviation error bars. The grouped bar chart illustrates the differences across all reported metrics, while the line chart compares the reported mIoU values across configurations.

The ablation progression clarifies the distinct functional roles of each recalibration stage. Spatial response amplification alone yields a substantial improvement over the bare baseline, suggesting that floor plan parsing is fundamentally a structural localization task rather than a texture classification problem. The larger incremental gain from adaptive channel gating indicates that suppressing background-dominated feature channels is even more critical than spatial refinement for this domain, because architectural drawings contain large empty regions that easily dilute semantic signals. When both mechanisms are combined, the synergy exceeds their individual contributions: spatial gating limits background noise entering the skip connections, while channel gating keeps the remaining spatial cues semantically relevant. The final addition of ADPE further improves overall performance, not by correcting gross errors but by refining the geometric alignment of rooms that share long parallel walls or symmetric layouts. Removing SGP leaves region overlap nearly unchanged (71.3 vs. 71.0 mIoU) but more clearly reduces Boundary F1 and Boundary IoU, consistent with its role as a boundary-level regularizer. This hierarchical pattern of improvement—first semantic, then spatial, finally coordinate-aware—is consistent with a geometry-first view of floor plan parsing.

To analyze the source of overall improvement, Table 4 reports the room-branch per-class IoU.

**Figure 7:**
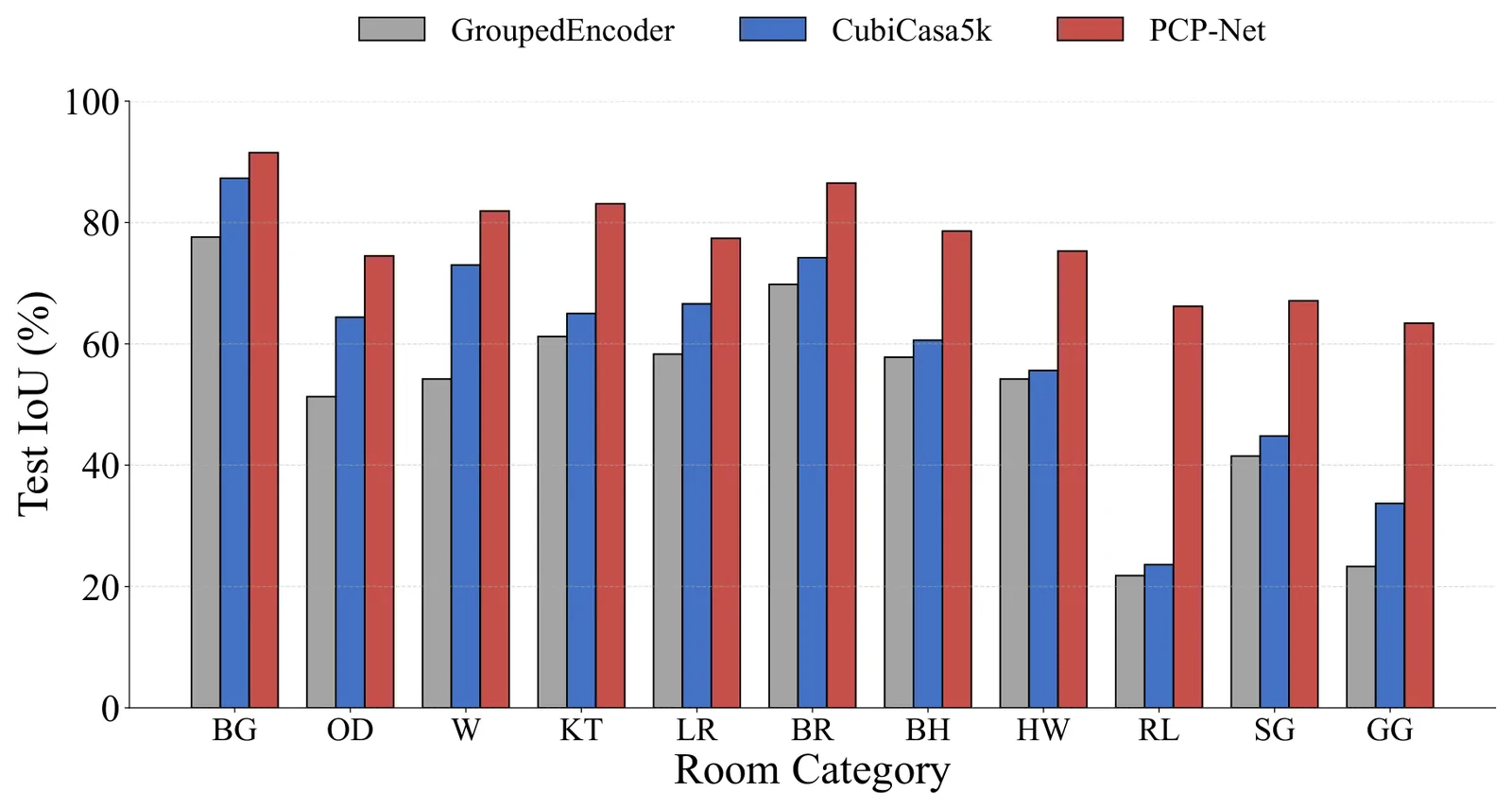
Room-branch per-class Test IoU comparison among the grouped encoder baseline, the CubiCasa5K baseline, and the full PCP-Net. The proposed recalibration modules recover structurally challenging categories such as Hallway, Railing, and Garage.

Figure 7 visualizes the per-class recovery, which is consistent with the largest gains occurring in spatially sensitive and tail categories.

The room-branch per-class breakdown reveals that the baseline architecture copes reasonably with large, centrally located rooms such as living rooms and bedrooms, where the receptive field naturally captures the full extent of the region. However, it performs substantially worse on tail categories that depend on structural context rather than interior texture: hallways are frequently absorbed into adjacent rooms because the model lacks awareness of their connecting role; railings are misclassified as walls because both share line-like features without positional disambiguation; and garages are confused with outdoor spaces due to their similar floor texture and peripheral location. After introducing ACG and SRA, the largest absolute gains occur precisely in these structurally challenging categories, which is consistent with recalibration restoring the boundary cues that define such spaces. ADPE further elevates spatially sensitive classes—hallways, railings, and storage—which may be explained by their dependence on knowing whether a region lies on a circulation path or at the plan periphery, which is exactly the coordinate-aware information that axis-decomposed embedding preserves.

Table 5 reports the icon-branch per-class IoU.

The icon branch exhibits a similar pattern, with the largest gains in small-symbol categories. Small symbols such as chimneys, bathtubs, and fireplaces are rarely detected by the baseline, not because they occupy few pixels but because their weak texture contrast is drowned out by the surrounding wall lines during encoder downsampling. The intermediate configuration recovers most of these categories by amplifying spatial peaks and suppressing background channel responses, yet some symbols remain partially fragmented or merged with nearby doors. The remaining ADPE-related gains are modest (at most 1.2 points per class); a plausible explanation is positional reasoning: bathtubs are usually located against bathroom walls and fireplaces on the exterior wall of living rooms, so ADPE may help the decoder resolve ambiguous symbols through contextual positioning rather than local appearance alone. This suggests that for architectural parsing, coordinate-aware modulation acts as an additional positional cue that becomes useful when visual cues alone are insufficient for disambiguation.

## 5. Discussion

The proposed architecture is motivated by the structural characteristics of architectural drawings. Grouped residual transformations increase local pattern diversity, which is useful for handling different wall widths, symbol shapes, and drawing conventions. CSFR improves skip connections by transforming them from raw high-resolution features into recalibrated structural features. ADPE is particularly suited to architectural drawings because many semantic cues follow horizontal and vertical axes. SGP complements feature recalibration by directly regularizing boundary gradients, and its contribution is evaluated through the boundary-level metrics and the ablation study in Section 4.5.

Despite these advantages, several limitations remain. First, extremely complex layouts, non-Manhattan structures, curved walls, and large open-plan spaces may still produce ambiguous boundaries. Second, heavily blurred scans or hand-drawn sketches may violate the assumptions of clean line structures. Third, zero-shot cross-domain evaluation is restricted to the overlapping categories of each target dataset and may be affected by inconsistent annotation granularity across datasets. Fourth, the boundary-level metrics (Boundary F1 and Boundary IoU) are proxy measures of boundary alignment rather than a complete assessment of topological correctness. Finally, the current framework focuses on two-dimensional semantic parsing and does not directly generate BIM objects; downstream BIM-related workflows require additional vectorization, topology graph construction, and height or material attribution.

Regarding the relation to BIM, the segmentation output of PCP-Net serves as an upstream semantic layer: room, icon, and boundary maps can feed vectorization and topology-graph construction, from which three-dimensional building models can be extruded and augmented with attribute data. PCP-Net itself does not perform these downstream steps, which remain outside the scope of this study.

Future work will explore multimodal floor plan understanding by integrating OCR and visual parsing, joint optimization with vectorized output generation, and improved modeling for non-Manhattan layouts. Transformer-based global modeling may also be combined with the proposed axis-aware recalibration when sufficient training data are available. We also plan to release the evaluation scripts and category-mapping files to facilitate reproducible benchmarking.

## 6. Conclusions

This paper presents PCP-Net, a cross-scale feature recalibration network for architectural floor plan parsing. PCP-Net combines a Grouped Residual Encoder, a CSFR pipeline with ACG, SRA, and ADPE, and an SGP branch for boundary-aware regularization. These components jointly address semantic dilution, structural misalignment, and boundary fragmentation in floor plan segmentation.

The experiments evaluate PCP-Net on CubiCasa5K and three cross-domain datasets. PCP-Net achieves mIoU of 71.3%, OA of 90.1%, and MA of 78.9% on CubiCasa5K, with stable cross-domain results on R3D, CVC-FP, and ROBIN. Ablation studies support the contribution of the recalibration modules (ACG, SRA, ADPE), while the SGP branch is evaluated as a training-time boundary regularizer through boundary-level metrics.

PCP-Net provides a semantic parsing layer that may support downstream BIM-related workflows, intelligent architectural review, and large-scale digital transformation of legacy floor plan documents when combined with vectorization, topology graph construction, and attribute extraction. Future extensions will consider text–vision fusion, vectorized output generation, and stronger generalization to non-Manhattan or hand-drawn layouts. 

## Figures and Tables

**Table 1:** Main results on CubiCasa5k test set.

Method	OA	MA	Room mIoU	Icon mIoU	Boundary F1	Boundary IoU
U-Net	71.80	58.40	48.90	43.60	55.80	38.70
U-Net + ResNeXt	74.10	61.20	51.91	46.54	58.40	44.20
CubiCasa5K baseline	82.70	69.80	58.98	55.70	63.80	46.84
DeepLabV3+	85.60	71.40	62.80	60.10	66.20	49.48
PSPNet	84.90	70.20	61.30	58.60	65.10	48.26
PCP-Net	90.10	78.90	76.86	72.65	76.60	64.20

**Table 2:** Room-segmentation results on cross-domain datasets. NA indicates categories not annotated in the corresponding dataset.

Dataset	Approach	Acc.	W	DW	BH	LR	BR	H	BY	CT	IoU
R3D	TF2DeepFloorplan	0.90	0.98	0.83	0.78	0.93	0.79	0.68	0.49	0.54	0.66
R3D	DeepLabV3+	0.83	0.93	0.60	0.57	0.90	0.40	0.44	0.00	0.05	0.44
R3D	PSPNet	0.81	0.91	0.54	0.50	0.89	0.40	0.23	0.11	0.09	0.41
R3D	PCP-Net w/o ADPE	0.94	0.99	0.87	0.83	0.96	0.83	0.74	0.61	0.62	0.73
R3D	PCP-Net	0.95	0.99	0.88	0.84	0.96	0.84	0.75	0.62	0.63	0.74
CVC-FP	TF2DeepFloorplan	0.84	0.95	0.79	0.73	0.91	0.65	0.67	0.52	NA	0.62
CVC-FP	DeepLabV3+	0.81	0.89	0.61	0.54	0.87	0.47	0.49	0.35	NA	0.46
CVC-FP	PSPNet	0.74	0.86	0.57	0.42	0.79	0.41	0.39	0.33	NA	0.40
CVC-FP	PCP-Net w/o ADPE	0.91	0.97	0.90	0.84	0.95	0.74	0.79	0.67	NA	0.71
CVC-FP	PCP-Net	0.92	0.97	0.90	0.85	0.95	0.75	0.80	0.68	NA	0.72
ROBIN	TF2DeepFloorplan	0.92	0.93	0.86	0.82	0.93	0.85	NA	NA	NA	0.83
ROBIN	DeepLabV3+	0.88	0.90	0.83	0.80	0.89	0.82	NA	NA	NA	0.81
ROBIN	PSPNet	0.83	0.86	0.79	0.76	0.80	0.77	NA	NA	NA	0.78
ROBIN	PCP-Net w/o ADPE	0.97	0.99	0.92	0.89	0.98	0.90	NA	NA	NA	0.87
ROBIN	PCP-Net	0.97	0.99	0.93	0.90	0.98	0.91	NA	NA	NA	0.88

**Table 3:** Overall ablation results of PCP-Net components (mean ± std over three seeds).

Method	SGP	OA	MA	mIoU	BF1	BIoU
Baseline	Yes	74.1 ± 0.3	61.2 ± 0.4	52.9 ± 0.5	58.4 ± 0.6	44.2 ± 0.6
+SRA	Yes	84.2 ± 0.2	72.6 ± 0.3	62.7 ± 0.4	68.9 ± 0.5	55.3 ± 0.5
+ACG	Yes	86.3 ± 0.3	75.2 ± 0.4	66.3 ± 0.4	71.5 ± 0.5	58.1 ± 0.6
w/o ADPE	Yes	89.4 ± 0.2	78.1 ± 0.3	70.5 ± 0.3	74.8 ± 0.4	62.4 ± 0.5
w/o SGP	No	87.9 ± 0.2	77.6 ± 0.3	71.0 ± 0.3	75.6 ± 0.4	63.1 ± 0.5
PCP-Net	Yes	90.1 ± 0.2	78.9 ± 0.3	71.3 ± 0.3	76.6 ± 0.4	64.2 ± 0.5

**Table 4:** Room-branch per-class IoU on the CubiCasa5K test set.

Approach	Split	BG	OD	W	KT	LR	BR	BH	HW	RL	SG	GG
Grouped Encoder Baseline	Test	77.6	51.3	54.2	61.2	58.3	69.8	57.8	54.2	21.8	41.5	23.3
CubiCasa5K baseline	Test	87.3	64.4	73.0	65.0	66.6	74.2	60.6	55.6	23.6	44.8	33.7
PCP-Net w/o ADPE	Test	90.3	73.2	80.6	81.8	76.1	85.3	77.1	74.1	64.1	65.5	61.5
PCP-Net	Test	91.5	74.5	81.9	83.1	77.4	86.5	78.6	75.3	66.2	67.1	63.4

**Table 5:** Icon-branch per-class IoU on the CubiCasa5K test set.

Approach	Split	EM	WI	DR	CT	EA	TO	SK	SB	FP	BT	CH
Grouped Encoder Baseline	Test	85.1	56.5	47.2	57.3	56.8	56.2	43.1	61.9	29.1	10.2	8.5
CubiCasa5K baseline	Test	97.6	66.8	53.6	69.2	66.0	62.8	55.7	67.3	36.2	26.7	11.2
PCP-Net w/o ADPE	Test	98.1	77.4	71.6	80.3	80.1	76.2	67.1	75.1	60.3	51.8	52.8
PCP-Net	Test	98.3	78.1	72.4	80.9	80.9	76.8	68.3	75.9	61.4	52.5	53.6

## Data Availability

The datasets referenced in this study are publicly available or subject to the access policies of their original providers.

## Abbreviations

The following abbreviations are used in this manuscript:

ACG	Adaptive Channel Gating
ADPE	Axis-Decompositional Positional Embedding
BIM	Building Information Modeling
CSFR	Cross-Scale Feature Recalibration
MA	Mean Accuracy
mIoU	Mean Intersection over Union
OA	Overall Accuracy
PCP-Net	Planar Component Parsing Network
SGP	Structural Gradient Propagation
SRA	Spatial Response Amplifier
